# Frequency Shaping-Based Control Framework for Reducing Motion Sickness in Autonomous Vehicles

**DOI:** 10.3390/s25030819

**Published:** 2025-01-29

**Authors:** Soomin Lee, Chunhwan Lee, Chulwoo Moon

**Affiliations:** 1 Department of Future Mobility Convergence, Chonnam National University, Gwangju 61186, Republic of Korea; su393@jnu.ac.kr; 2 Department of Intelligent Mobility, Chonnam National University, Gwangju 61186, Republic of Korea; chunhwan@jnu.ac.kr

**Keywords:** autonomous vehicle, motion sickness, band-stop filter, ride comfort, path-following

## Abstract

This study introduces a motion-sickness-reducing control strategy aimed at enhancing ride comfort in Electric Autonomous Vehicles (EAVs). For lateral control, the forward look-ahead distance was adaptively adjusted based on the Motion Sickness Dose Value (MSDV) analysis from ISO 2631-1, effectively mitigating lateral acceleration and its motion-sickness-related frequency components, leading to a reduced MSDV. For longitudinal control, Linear Quadratic Regulator (LQR) optimal control was applied to minimize acceleration, complemented by a band-stop filter specifically designed to attenuate motion-sickness-inducing frequencies in the acceleration input. The bandwidth of the band-stop filter used in this study was designed based on the motion-sickness frequency weighting specified in ISO 2631-1. The simulation results of the proposed control indicate a significant reduction in MSDV, decreasing from 16.3 to 10.46, achieving up to a 35.8% improvement compared to comparative control methods. While the average lateral position error was slightly higher than that of the comparative controller, the vehicle consistently maintained lane adherence throughout path-following tasks. These findings underscore the potential of the proposed method to simultaneously mitigate motion sickness and achieve a robust path-following performance in autonomous vehicles.

## 1. Introduction

Electric Autonomous Vehicles (EAVs) have become a cornerstone of future transportation systems, propelled by recent technological advancements. These vehicles are equipped with sophisticated systems capable of autonomously planning routes and perceiving their surroundings, ensuring safe operation without human intervention. Significant efforts have been devoted to enhancing the path-following performance of autonomous vehicles. For instance, deep learning techniques have been leveraged to improve path-following capabilities [1], and reinforcement learning approaches have enabled vehicles to autonomously learn and navigate complex paths [2]. Moreover, model predictive control (MPC) has been applied to ensure accurate path-following, even on intricate and winding roads [3]. However, while these advancements have improved the path-following performance, they have also intensified the vehicle’s dynamic behavior, potentially compromising ride comfort.

To address motion sickness, several studies have explored methods such as enhancing the environment or providing passengers with directional information. For example, some studies predict the likelihood of motion sickness based on upcoming curves and notify passengers in advance [4], while others use onboard audio systems to improve passengers’ ability to anticipate vehicle movements [5]. Additionally, vibration patterns have been proposed to warn passengers of vehicle dynamics [6,7,8], and display systems have been designed to reduce sensory mismatches by providing real-time environmental information [9,10]. Virtual Reality (VR) technologies have also been explored to alleviate motion sickness in passengers [11]. These approaches primarily focus on reducing sensory conflicts by offering external cues. However, they often require passengers to pay close attention, potentially leading to inconvenience and restrictions on freedom of movement.

In contrast, approaches directly addressing motion sickness through vehicle control remain underexplored, particularly in autonomous vehicles. Active suspension systems have been utilized to reduce motion sickness by controlling vertical, pitch, and roll dynamics [12,13,14,15], while optimization methods such as Particle Swarm Optimization (PSO) have been used to minimize passenger discomfort [16]. Despite these efforts, studies focusing on dynamic control tailored for autonomous vehicles are still relatively sparse.

Unlike conventional vehicles, autonomous vehicles lack a driver, making passengers more sensitive to vehicle motions and increasing the likelihood of motion sickness. This condition primarily arises from sensory conflicts between vestibular inputs and visual information, with the highest sensitivity occurring at a low frequency of 0.16 Hz [17]. The severity of motion sickness is influenced by the magnitude, frequency, and duration of acceleration exposure.

To address these challenges, this study proposes a novel motion-sickness-mitigating control strategy for autonomous vehicles. The proposed approach integrates lateral control using a variable look-ahead distance (LAD), derived from a motion sickness analysis of the forward look-ahead distance in the Pure Pursuit controller, with longitudinal control combining a Linear Quadratic Regulator (LQR) and a band-stop filter to suppress accelerations in the critical 0.16 Hz frequency range. Unlike existing methods that rely on external cues, this strategy directly controls the vehicle’s dynamic behavior, offering a unique solution for alleviating motion sickness.

The effectiveness of the proposed algorithm was validated through simulations conducted in MATLAB/Simulink 2024B and Carmaker (IPG Automotive, Karlsruhe, Germania) environments, with the Motion Sickness Dose Value (MSDV) specified in ISO 2631-1 used as the evaluation metric [17]. The structure of this paper is as follows: Section 2 reviews motion sickness evaluation methods, Section 3 presents the proposed control framework, and Section 4 describes the simulation environment and validation results. Finally, Section 5 discusses the findings and future research directions while providing the conclusion.

## 2. Motion Sickness Evaluation

Motion sickness (MS) is a critical factor influencing ride comfort, arising primarily from discrepancies between expected and actual motion. Passengers are significantly more susceptible to motion sickness compared to drivers. In autonomous vehicles, where all occupants are passengers, the likelihood of motion sickness increases dramatically. A recent study highlights that approximately two-thirds of autonomous vehicle passengers are expected to experience motion sickness, posing a significant barrier to widespread adoption and commercialization of autonomous vehicles [18].

Evaluation methods for motion sickness include psychological surveys, physiological signal measurements, and the Motion Sickness Dose Value (MSDV). MSDV provides a robust and quantitative measure of motion sickness, making it widely applicable for evaluating the effectiveness of mitigation strategies. ISO 2631-1 primarily evaluates motion sickness in the vertical (Z-axis) direction using frequency weighting. However, prior research has demonstrated that horizontal (X, Y-axis) motion plays a dominant role in inducing motion sickness in vehicles, surpassing the effects of vertical (Z-axis) acceleration [19]. Notably, horizontal motion induces more severe symptoms than vertical motion, with the greatest sensitivity occurring at frequencies below 0.2 Hz [20,21]. This finding underscores the need for control strategies that specifically address horizontal motion to improve passenger comfort.

Despite its importance, addressing motion sickness in autonomous vehicles through direct vehicle control remains a complex challenge. Horizontal (X, Y-axis) accelerations are inherently more difficult to predict and control due to the variability in driving conditions and the dynamic nature of autonomous systems. These challenges necessitate innovative control strategies capable of reducing motion sickness while maintaining overall vehicle performance.

Based on these insights, this study investigates a control method to reduce MSDV in autonomous vehicles by applying frequency weighting to horizontal (X, Y-axis) accelerations. To quantitatively assess motion sickness, the MSDV is computed as follows:

(1) $$MSDV={\left\{\int_{0}^{T}{\left[a_{w}\left(t\right)\right]}^{2dt}\right\}}^{\frac{1}{2}}a$$

where $a_{xw}$ and $a_{yw}$ represent the $W_{f}$ frequency weighted longitudinal and lateral accelerations, respectively, and T denotes the driving duration. This study quantitatively evaluates motion sickness based on MSDV.

The factors influencing MSDV (Motion Sickness Dose Value) include acceleration, the frequency of acceleration, and travel time. Lateral acceleration and the frequency of lateral acceleration are expected to vary depending on the settings of the path-following controller, which could influence motion sickness. Similarly, longitudinal acceleration and the frequency of longitudinal acceleration, caused by acceleration and deceleration during speed control, are also anticipated to affect motion sickness. This paper proposes a method to reduce vehicle acceleration and the motion sickness-related frequency of acceleration through control strategies aimed at mitigating MSDV.

## 3. Motion Sickness Reduction Control Algorithm

**Figure 1:**
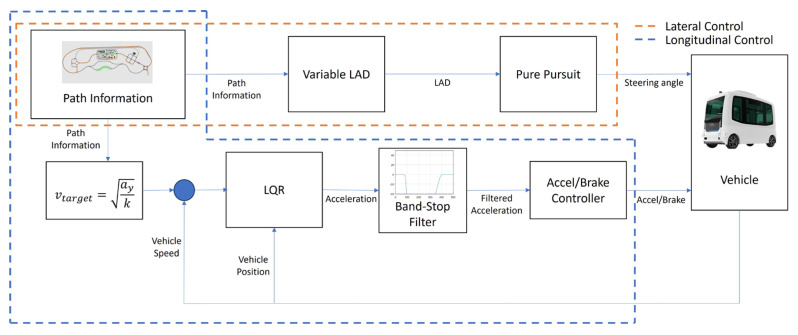
Motion sickness reduction control diagram.

In this study, the Pure Pursuit controller, which exhibits variations in path-following performance depending on the look-ahead distance setting, was analyzed to evaluate MSDV under different look-ahead distances. Based on the analysis results, a motion-sickness-mitigating variable look-ahead distance is proposed. Additionally, by combining LQR optimal control with a band-stop filter, longitudinal acceleration and longitudinal acceleration components within the motion-sickness-sensitive frequency range are reduced, ultimately lowering the MSDV. The overall framework of the motion sickness reduction control is illustrated in Figure 1.

### 3.1. Lateral Control

#### 3.1.1. Pure Pursuit Algorithm

In this study, it is anticipated that both the lateral acceleration and the frequency of the lateral acceleration will vary depending on the path-following control. To analyze this, the Pure Pursuit algorithm, which significantly affects path-following performance based on the look-ahead distance settings, was utilized to conduct motion sickness analysis according to variations in the look-ahead distance.

The Pure Pursuit algorithm is a geometric path-tracking method that follows a target point on a predefined path. This target point is located within a configurable look-ahead distance (LAD) from the rear axle center of the vehicle. The algorithm is widely used due to its simplicity and effectiveness in achieving smooth path-following.

The foundation of the Pure Pursuit algorithm lies in the Ackermann steering angle. The Ackermann steering angle, derived from the bicycle model, describes the relationship between the front wheel steering angle and the turning radius. This relationship assumes low-speed operation where tire–road contact is constrained, and tire slip does not occur. The steering angle and the turning radius are related as follows:

(2) $$\tan\left(\delta \right)=\frac{L}{R}$$

(3) $$\delta ={tan}^{-1}\left(\frac{L}{R}\right)$$

Here, *L* represents the wheelbase, and *R* denotes the turning radius. These equations form the basis for the geometric steering model.

**Figure 2:**
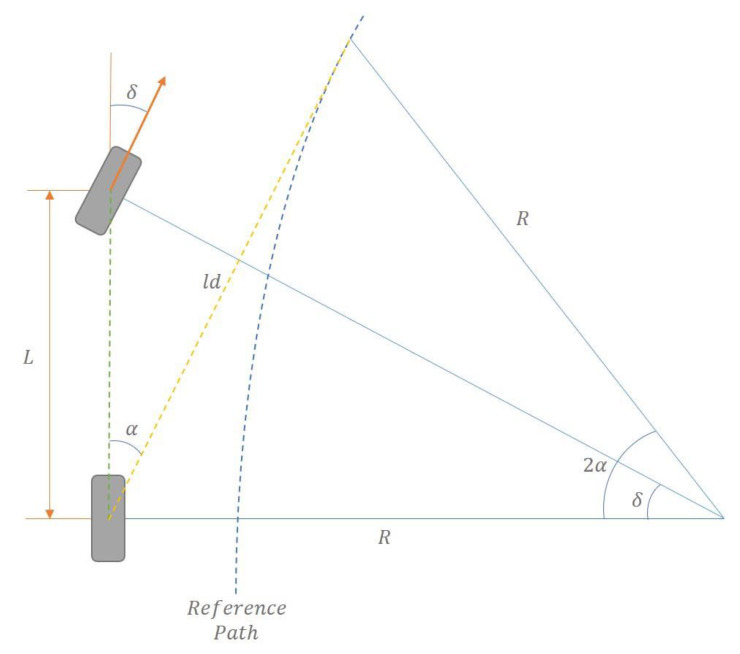
Pure Pursuit algorithm.

The derivation of the Pure Pursuit algorithm is shown in Figure 2 and involves the following geometric relationships:

(4) $$\sin\left(\alpha \right)=\frac{e}{l_{d}}\left(\frac{L}{R}\right)$$

(5) $$\frac{\sin\left(\alpha \right)}{l_{d}}=\frac{sin\left(\frac{\pi }{2}-\alpha \right)}{R}\left(\frac{L}{R}\right)$$

Here, α represents the angle between the rear axle and the predicted target point, $l_{d}$ denotes the look-ahead distance, and *e* represents the cross-track error, which is the perpendicular distance between the vehicle’s current position and the target path. By integrating these relationships with the Ackermann steering angle Equation (3), the Pure Pursuit algorithm is reduced to the following final form:

(6) $$\tan\left(\delta \right)=\frac{2L\;\sin\left(\alpha \right)}{l_{d}}$$

(7) $$\delta \left(PP\right)={tan}^{-1}\left(\frac{2*L*sin(\alpha \left(t\right))}{l_{d}}\right)$$

This equation demonstrates how the look-ahead distance influences the steering angle, enabling smooth and stable path tracking along curved paths.

**Figure 3:**
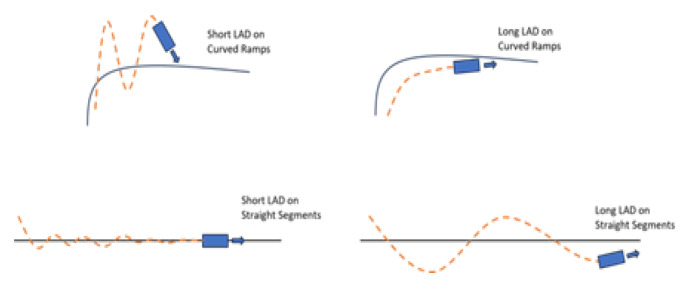
Changes in vehicle dynamics according to look-ahead distance settings.

The choice of look-ahead distance is a critical factor in the performance of the Pure Pursuit algorithm. A shorter look-ahead distance increases sensitivity to small path deviations, potentially causing oscillations and unstable driving. Conversely, a longer look-ahead distance reduces sensitivity, which may result in corner cutting and imprecise path tracking. Therefore, selecting an optimal look-ahead distance is essential for balancing smoothness and accuracy in path-following performance. The changes in vehicle dynamics according to look-ahead distance settings are illustrated in Figure 3.

This study aimed to analyze the acceleration and acceleration frequency associated with the look-ahead distance setting of the Pure Pursuit algorithm, which exhibit significantly different behaviors, and to propose a motion-sickness-reducing look-ahead distance.

#### 3.1.2. Motion Sickness Analysis Based on Look-Ahead Distance

**Figure 4:**
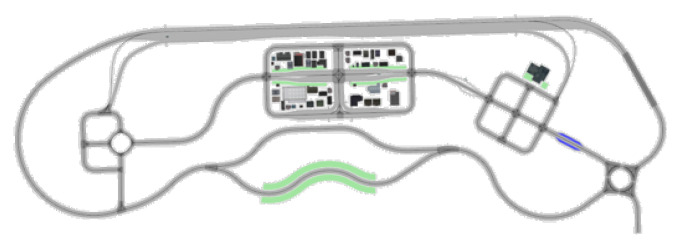
Simulation test routes in K-City.

To analyze the effects of look-ahead distance (LAD) variations on motion sickness in the Pure Pursuit algorithm, a simulated road network was constructed using high-precision maps of the K-City autonomous vehicle testing facility provided by the Korea Automobile Testing and Research Institute (KATRI). The simulation test routes in K-City are shown in Figure 4. The vehicle used in the simulations was the KAMO-U autonomous shuttle, developed by the Korea Automotive Technology Institute (KATECH, Cheonan-Si, Republic of Korea). All analyses were conducted at speeds of up to 40 kph.

**Figure 5:**
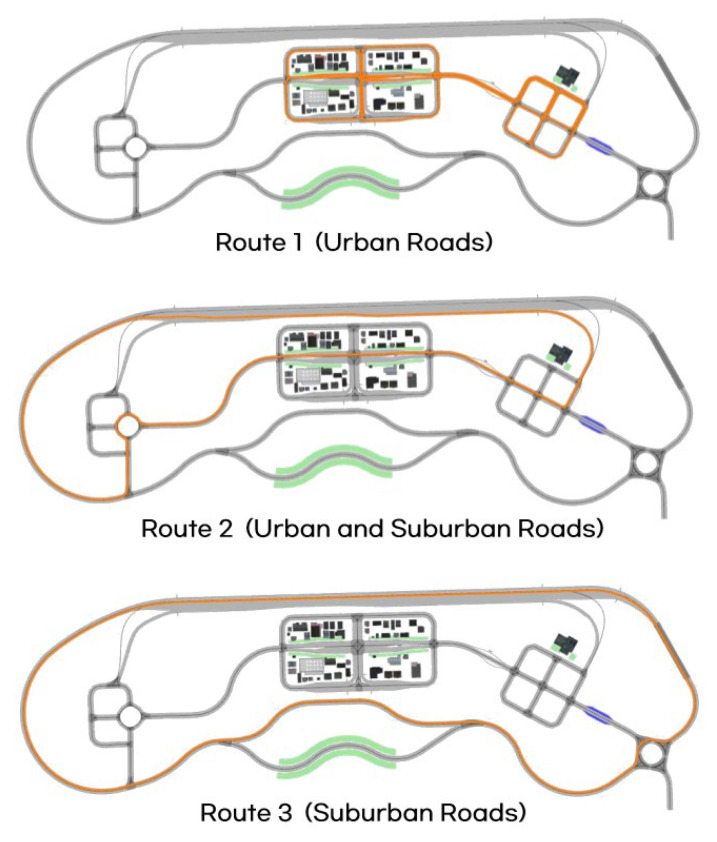
Three routes in K-City for motion sickness analysis (Route 1, Route 2, Route 3).

For the analysis of Motion Sickness Dose Value (MSDV), three routes were selected as shown in Figure 5: Route 1 represents urban roads, Route 2 includes both urban and suburban roads, and Route 3 consists entirely of suburban roads. For each route, the frequency-weighted acceleration, peak-to-peak acceleration, and MSDV were evaluated under varying LAD settings.

Three types of routes were selected for the MSDV analysis based on varying look-ahead distances, as shown in Figure 5: Route 1 represents urban roads, Route 2 includes both urban and suburban roads, and Route 3 consists entirely of suburban roads. For each route, the frequency weighted acceleration, peak-to-peak acceleration, and MSDV were evaluated under different look-ahead distances.

The results showed that increasing the look-ahead distance reduced the dominant frequency weighting and the peak-to-peak acceleration.

The analysis results showed that increasing the LAD significantly reduced both the dominant frequency weighting and the peak-to-peak acceleration. In the Pure Pursuit algorithm, the LAD is a critical parameter for determining the steering angle by considering the target point relative to the vehicle’s current position. A longer LAD allows the vehicle to anticipate path changes earlier, enabling smoother steering inputs and improved path-following performance.

**Figure 6:**
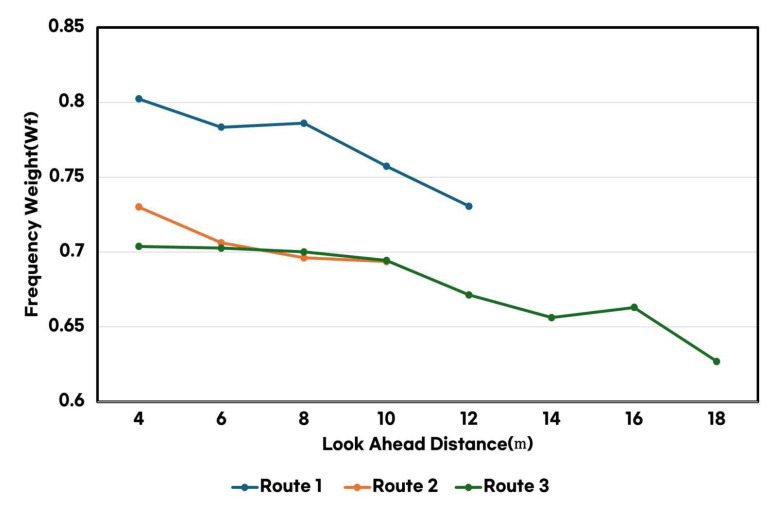
Frequency weighting comparison based on look-ahead distance across the three routes.

As shown in Figure 6, increasing the LAD effectively reduced the motion-sickness-inducing frequency components of lateral acceleration. This reduction in frequency components led to a significant decrease in the frequency weighting values ($w_{f}$), thereby contributing to an overall reduction in MSDV. Specifically, the smoother trajectory generated by a longer LAD effectively mitigated vibrations within the critical frequency range associated with motion sickness.

**Figure 7:**
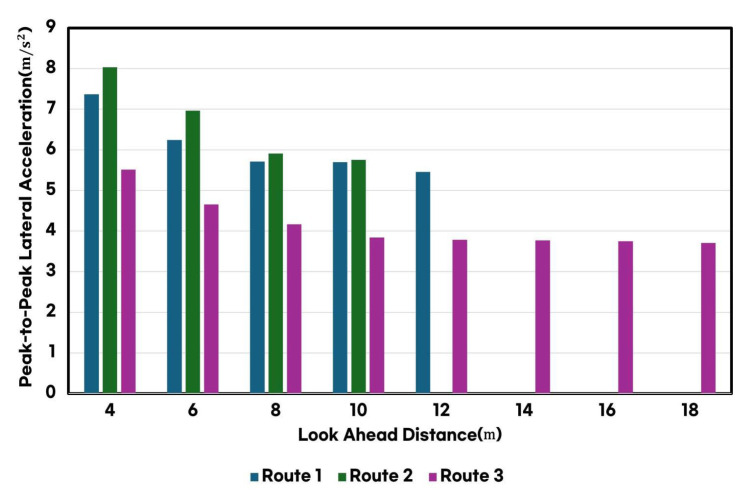
Comparison of peak-to-peak lateral acceleration based on look-ahead distance across the three routes.

Additionally, as illustrated in Figure 7, the LAD had a direct impact on lateral acceleration. A longer LAD reduced abrupt steering inputs, significantly decreasing both the maximum values and the variation rates of lateral acceleration. This reduction in lateral acceleration magnitude lowered the frequency-weighted acceleration used in the MSDV calculation, ultimately leading to a substantial decrease in MSDV.

These findings indicate that LAD can serve as a crucial control parameter for minimizing motion sickness. Furthermore, by appropriately adjusting the LAD, it is possible to effectively manage the trade-off between path-following performance and motion sickness mitigation. Future research should explore optimal LAD adjustment strategies under various road conditions and driving scenarios to further enhance the balance between ride comfort and path-following accuracy.

#### 3.1.3. Motion Sickness Reducing Variable Look-Ahead Distance

**Figure 8:**
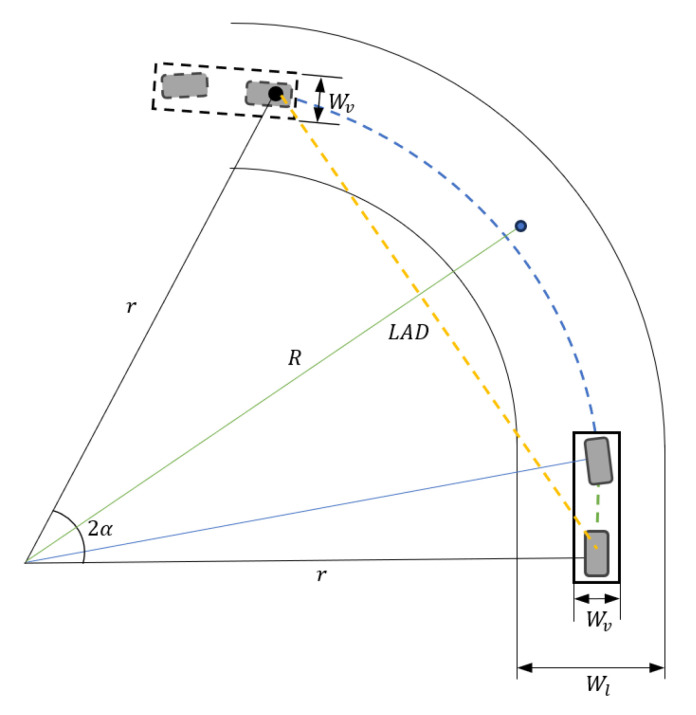
Variable look-ahead distance diagram for motion sickness mitigation.

The MSDV analysis demonstrated that a longer look-ahead distance consistently resulted in lower MSDV values. Based on this finding, a variable look-ahead distance was employed to reduce motion sickness. The variable look-ahead distance diagram for motion sickness mitigation is illustrated in Figure 8. To ensure that the vehicle remained within lane boundaries, a regression analysis was conducted to determine the maximum allowable look-ahead distance while considering the vehicle’s lane width and cross-track error.

To derive the variable look-ahead distance that prevents lane departure, the relationship between the turning radius, steering angle, and cross-track error (CTE) was analyzed. The turning radius *R* is expressed as follows:

(8) $$R=\frac{LAD}{2\;\sin(\alpha )}$$

Using this turning radius, the cross-track error is calculated as follows:

(9) CTE=R−(r−Max CTE) 

where *r* is the vehicle’s lateral position relative to the lane center, and Max CTE is the maximum allowable lateral offset.

The maximum cross-track error is constrained by the lane width ($W_{l}$) and vehicle width ($W_{v}$) as follows:

(10) $$Max\;CTE=(\frac{W_{l}-W_{v}}{2})=0.88m\;$$

Based on these constraints, a linear regression model for the maximum look-ahead distance ($L_{d}$) was developed:

(11) $${\text{Linear fit}:L}_{d}=0.2118*k+6.14\;$$

Additionally, a quadratic regression model was derived for greater accuracy:

(12) $${\text{Quadratic Fit}:L}_{d}=-0.0013*k^{2}+0.3539*k+3.2983$$

**Figure 9:**
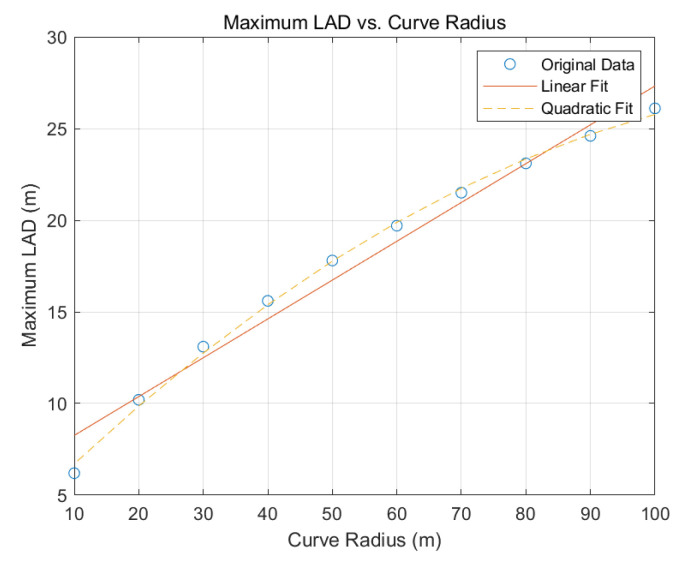
Maximum look-ahead distance results for linear regression and quadratic regression.

The results for the maximum look-ahead distance from the linear and quadratic regression analyses are shown in Figure 9. The linear regression analysis demonstrated high reliability, with a coefficient of determination ($R^{2}$) of 0.9734. This high $R^{2}\;$ value reflects the simulation’s fidelity and the minimal tire slip observed at the maximum speed of 40 kph. Given its high reliability and suitability for real-time computation, the linear regression model was ultimately selected for implementation.

The calculated look-ahead distance was set with a 10% safety margin for stability, with a maximum look-ahead distance of 20 m in straight sections and a minimum of 5 m in curved sections. This value was determined based on the curvature radius and the vehicle’s wheelbase. The variable look-ahead distance control dynamically adapts to the curvature radius. In the simulation environment, the current position and the set preview distance are used with GPS to obtain the curvature at that distance from the preloaded path information. Based on this curvature, the variable look-ahead distance control is applied, calculating the look-ahead distance in real time to prevent lane departures and reduce motion sickness. By utilizing cross-track error and lane width parameters, this control method strikes a balance between motion sickness mitigation and path-following performance.

For comparison, the control algorithm proposed by Kim et al., which uses speed-adaptive look-ahead distances [22], was implemented. The corresponding equation is given as follows (13). These findings demonstrate that LAD can be used as a control parameter to minimize motion sickness. This facilitates balancing the trade-off between path-following performance and passenger comfort.

(13) $$LAD=\left\{\begin{array}{cc} \begin{array}{c} \;5\;\\ \;0.8\; \end{array} & \begin{array}{c} \;\;\times \end{array}\\ 20 & \end{array}\right.\;v\;\;\;\;\begin{array}{c} \;\;\;\begin{array}{c} v\;\;\leq \;\;15\;kph\;\\ 15\;\mathrm{kph}\;<\;v\;\;<\;\;40\;\mathrm{kph}\; \end{array}\\ 40\;\mathrm{kph}\;\;\leq \;\;v \end{array}$$

Speed control was implemented using feedback and proportional control to ensure the target speed matched the curvature of the path. The MSDV comparison results for three paths, based on reference LAD and variable LAD, are presented in Table 1.

### 3.2. Longitudinal Control

**Figure 10:**
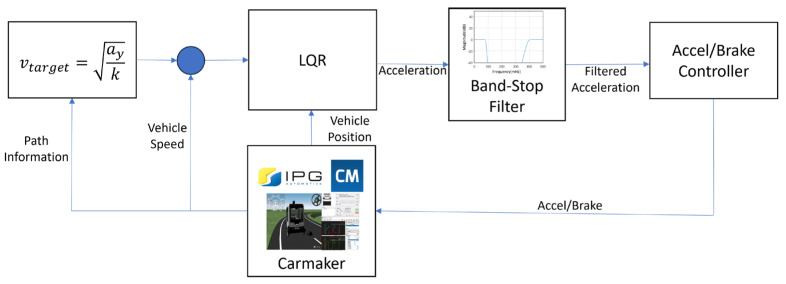
Longitudinal control diagram.

The longitudinal control aims to reduce the Motion Sickness Dose Value (MSDV) by minimizing acceleration and frequency-weighted acceleration, which are causally related to MSDV, during travel time. To achieve this, Linear Quadratic Regulator (LQR) optimal control is utilized to reduce longitudinal acceleration, and a band-stop filter is designed based on the frequency weighting function $W_{f}$ specified for MSDV. This filter attenuates the low-frequency components of acceleration within the motion-sickness-sensitive range. The filtered acceleration, which serves as the control input, effectively reduces motion sickness-inducing frequencies. The longitudinal control diagram is shown in Figure 10.

#### 3.2.1. LQR Speed Control

This study aims to reduce MSDV by minimizing acceleration through LQR optimal control-based speed regulation and mitigating accelerations in the motion sickness frequency range using a band-stop filter.

This study enhances the feasibility of real-time implementation by integrating constraints into LQR-based control. Prior research has addressed the constrained linear quadratic regulation problem for linear systems through approaches such as dynamic programming and partial quadratic function approximation techniques, as well as using model predictive control (MPC) to efficiently manage constraints and tackle complex control challenges [23,24]. However, this study employs LQR control due to its inherent simplicity, low computational cost, and suitability for real-time applications, particularly in autonomous vehicles.

Although handling constraints in traditional LQR may pose potential stability challenges in specific scenarios, the proposed approach strikes a practical balance between computational efficiency and implementation feasibility. This makes it particularly advantageous for dynamic and complex real-time driving environments, offering a robust and effective solution for the control of autonomous vehicles.

The state variable x is defined using position and speed, and the control input is defined as acceleration.

(14) $$\dot{x}=Ax+Bu\;$$

(15) $$x=[\;\;p\;\;v\;{]}^{T}\;$$

(16) $$A=\left[\begin{array}{cc} 0 & 1\\ 0 & 0 \end{array}\right],\;\;B={\left[\begin{array}{c} 0\\ 1 \end{array}\right]}^{T}\;\;\;$$

The control objective of the LQR controller is to find the optimal gain that minimizes the cost function defined in Equation (17).

(17) $$J=\int_{0}^{\infty }\left(x^{T}Q\;x+u^{T}R\;u\right)\;dt$$

The state weighting matrix Q and control weighting R represent the weights for the cost function and control input, respectively. The optimal gain that minimizes the cost function in Equation (18) can be determined by solving the Riccati equation.

(18) $$A^{T}P+PA-{PBR}^{-1}B^{T}P+Q=0\;$$

Using the solution P obtained from the Riccati equation, the optimal gain K is calculated as follows:

(19) $$K=R^{-1}B^{T}P$$

The control input u, which minimizes the cost function, is computed using the following formula. To prevent sudden acceleration or deceleration, an upper limit for $\pm \;1.5\;m/s^{2}$ is applied as shown in Equation (21).

(20) u=−Kx.

(21) u=max(−1.5,min(−Kx,  1.5)

#### 3.2.2. Band-Stop Filter Design

A band-stop filter was implemented to reduce the low frequency components of acceleration being generated during vehicle acceleration and deceleration. A Butterworth filter was employed as the band-stop filter, referencing the frequency weighting function $W_{f}$ defined in ISO 2631-1. The stopband was set between 0.08 Hz and 0.4 Hz. To implement the Butterworth filter as a band-stop filter, both a low-pass filter and a high-pass filter are utilized in combination. The mathematical expressions for the low-pass and high-pass filters are as follows:

(22) $$H_{lp}(jw)=\frac{1}{\sqrt{1+(\frac{\omega }{\omega_{c,lp}}{)}^{2n}}}\;$$

(23) $$H_{hp}(jw)=\frac{1}{\sqrt{1+(\frac{\omega_{c,hp}}{\omega }{)}^{2n}}}\;$$

**Figure 11:**
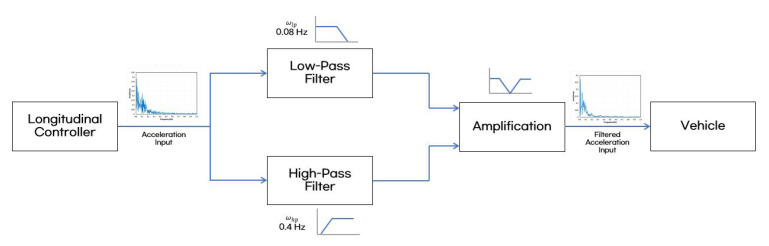
Diagram of longitudinal control with motion-sickness frequency reduction via acceleration filtering.

In this context, $w_{c}$ represents the cutoff frequency, and n is the filter order. The attenuation of the designed band-stop filter was set to 10 dB, which helped reduce motion sickness while preventing significant increases in travel time and avoiding sudden deceleration and acceleration. The diagram of longitudinal control with motion-sickness frequency reduction via acceleration filtering is presented in Figure 11.

#### 3.2.3. Target Speed Based on Road Curvature

The target speed of the vehicle was determined using the relationship among speed, curvature, and acceleration to ensure the lateral acceleration during cornering does not exceed 3 $m/s^{2}$, as specified in ISO 11270 for Lane Keeping Assistance Systems (LKASs). The target speed equation is as follows:

(24) $$V_{target}=\sqrt{\frac{a_{target}}{k}}\;$$

Here, $V_{target}$ is the target speed, $a_{target}$ is the target lateral acceleration, and k is the road curvature. By inputting the desired target lateral acceleration, the corresponding speed based on curvature can be determined.

According to a highway engineering study, drivers adjust their speed to maintain a lateral acceleration within the acceptable range of 0.2–0.4 g’s [25]. Another study indicates that drivers manage their acceleration or braking to ensure their cornering lateral acceleration stays within the range of 0.3–0.5 g’s [26]. In this study, based on the lateral acceleration typically experienced by general drivers and the lateral acceleration limit enforced by ISO 11270 for Lane Keeping Assistance Systems (LKAS), a limit of 3 m/s^2^ is applied. However, these values, along with the control parameters, damping, and bandwidth, can be adjusted based on individual or group requirements. Studies on personalized control strategies for different driving styles and real-world conditions address the possibility of adjusting these values. Research has highlighted the critical role of dynamically adjusting control parameters based on road conditions and driver preferences to improve the adaptability and performance of autonomous systems. Some studies have concentrated on utilizing machine learning and predictive modeling to understand and classify driving styles [27,28], while others have focused on integrating social and individual preferences into decision-making processes for specific scenarios, such as lane changes or urban intersections [29,30]. Furthermore, methodologies inspired by human decision-making frameworks have been proposed to address the complexities of mixed-traffic environments [31]. However, in this study, fixed values were used for comparison with the MSDV of the existing path-following controller. Future work will focus on fine-tuning and optimizing the control parameters to better match the driver’s style, enabling a more personalized control system.

## 4. Simulation and Result

### 4.1. Simulation Environment

To validate the proposed control method, a control environment was constructed using MATLAB/Simulink, and simulation tests were conducted using Carmaker. The test vehicle was the autonomous shuttle KAMO_U, with a maximum speed of 40 km/h. Detailed specifications of the simulation vehicle are summarized in Table 2.

**Figure 12:**
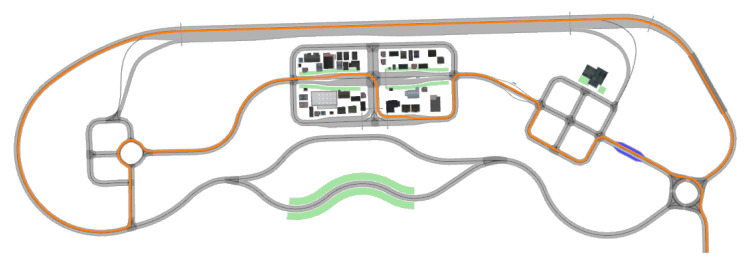
Composite K-city Route for MSDV evaluation.

The evaluation route was selected to include various conditions such as tight curves and frequent turns in community and urban areas of K-City, merging sections and narrow roads in suburban areas, and straight sections on highways, as shown in Figure 12.

### 4.2. Simulation Results

For comparison, a control system using PID-based speed control and the speed-adaptive look-ahead distance for the Pure Pursuit algorithm was employed. The simulation results, including MSDV, travel time, and maximum cross-track error, are summarized in Table 3. These results demonstrate the effectiveness of the proposed method in reducing motion sickness and maintaining lane stability across the evaluation routes. 

**Figure 13:**
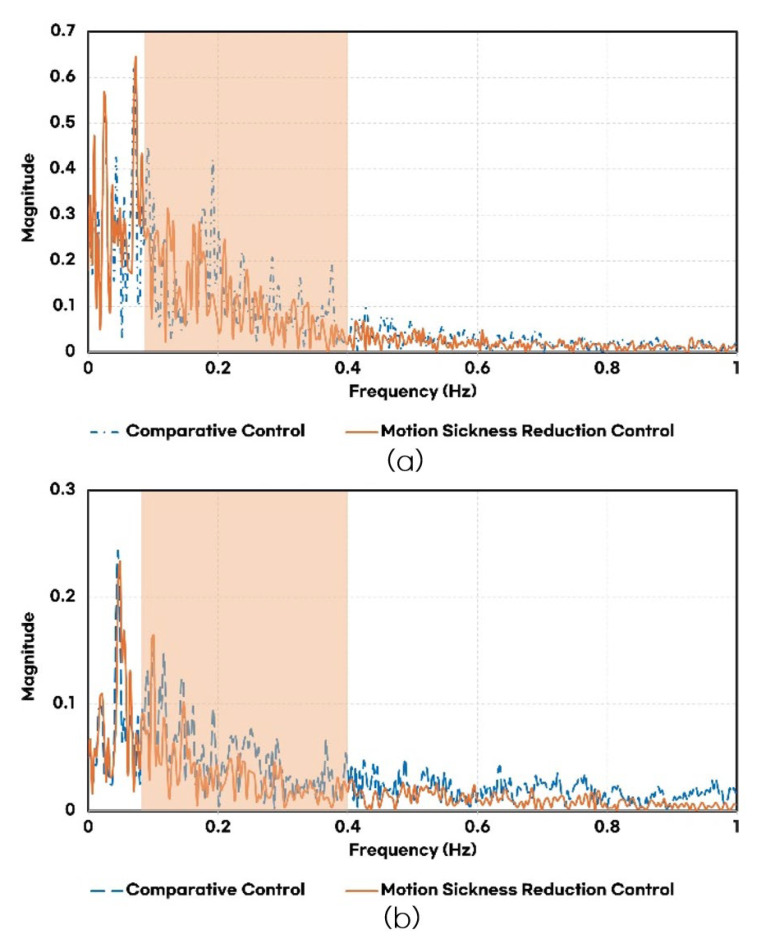
Frequency comparison for each controller: (**a**) frequency of lateral acceleration; (**b**) frequency of longitudinal acceleration.

Figure 13 compares the frequency components of lateral and longitudinal accelerations for the controllers. The motion-sickness-reducing variable look-ahead distance effectively reduced acceleration components in the frequency range associated with motion sickness. Similarly, LQR optimal control and the band-stop filter significantly suppressed motion-sickness-inducing frequency components in the longitudinal acceleration spectrum.

**Figure 14:**
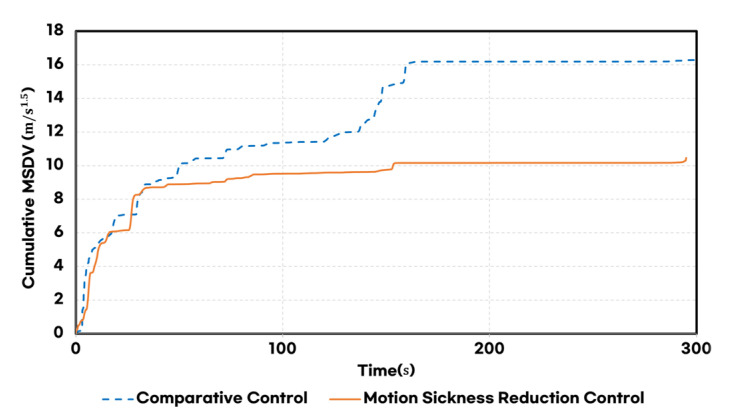
Comparison of MSDV for each controller.

As a result, the proposed method achieved a 35.8% reduction in MSDV compared to the baseline controllers, as shown in Figure 14.

**Figure 15:**
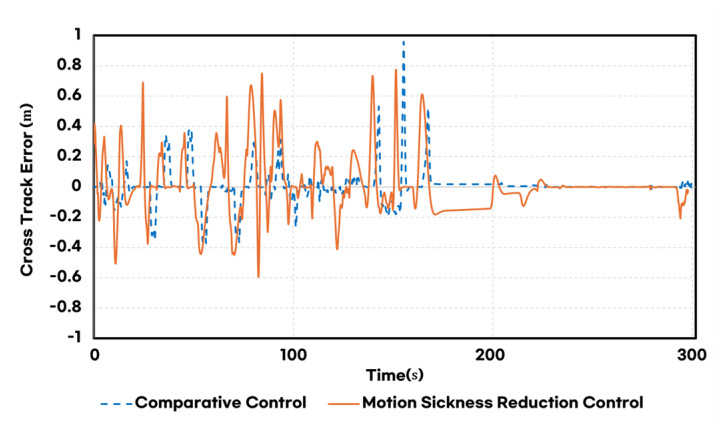
Comparison of cross-track error for each controller.

Figure 15 compares vertical path errors. Although the proposed control exhibited higher vertical path errors in curved sections than the baseline, the maximum vertical path error remained within 0.776 m, ensuring the vehicle stayed within the lane. In contrast, the baseline controller showed lower average vertical path errors but a higher maximum error of 0.96 m. This occurred in narrow radius rotary sections due to the lack of curvature consideration in the speed-adaptive look-ahead distance, leading to lane departure.

The proposed method demonstrated significant improvements in reducing MSDV and maintaining lane-keeping performance. However, several limitations must be addressed. First, while the use of the variable look-ahead distance effectively reduced MSDV, it also increased the average cross-track error, which may indicate potential risks in real-world driving conditions. Further optimization of the look-ahead distance is necessary to improve overall stability. Second, although the computational efficiency of the combined LQR and band-stop filter approach was validated in the given scenarios, additional evaluations under high-speed and highly dynamic conditions are required. Advanced control strategies, such as model predictive control (MPC) and predictive control, could be considered to enhance performance. Finally, real-world testing is essential to evaluate the effects of sensor noise, latency, and hardware limitations on the practical implementation of the proposed method.

## 5. Conclusions

This study presented an integrated control framework designed to enhance ride quality and path-following accuracy in Electric Autonomous Vehicles (EAVs) while mitigating motion sickness. The proposed approach combines lateral control using a variable look-ahead distance (LAD) in the Pure Pursuit algorithm and longitudinal control through Linear Quadratic Regulator (LQR) optimization integrated with a band-stop filter. By directly targeting motion-sickness-inducing accelerations in the frequency range defined by ISO 2631-1, this framework offers a practical solution to improve passenger comfort.

In lateral control, the adaptive adjustment of the LAD enabled a smoother path-following performance by reducing lateral acceleration and its motion-sickness-sensitive frequency components. This adjustment significantly decreased the Motion Sickness Dose Value (MSDV) while maintaining lane adherence. For longitudinal control, LQR optimization coupled with a band-stop filter effectively attenuated longitudinal accelerations within critical frequency ranges, balancing ride comfort and motion sickness mitigation.

Simulation results validated the effectiveness of the proposed framework, showing significant reductions in MSDV and suppressing motion-sickness-inducing frequency components in both lateral and longitudinal directions. The findings underscore the potential of this scalable and adaptable control strategy for autonomous PBVs, offering robust performance across diverse driving conditions.

1. The proposed control framework achieved a 35.8% reduction in MSDV, demonstrating its superior effectiveness compared to conventional control methods.
2. A frequency domain analysis confirmed substantial suppression of motion-sickness-inducing accelerations in both lateral and longitudinal directions.
3. The combination of variable LAD and LQR optimal control with a band-stop filter reduced MSDV while maintaining lane adherence, meeting the requirements for both motion sickness mitigation and path-following performance.

Future work will focus on optimizing and precisely tuning the control parameters, evaluating the performance under various traffic and road conditions, and validating the control strategy’s applicability through real-world road tests.

## Figures and Tables

**Table 1:** Comparison of MSDV Results for three routes: Variable LAD and Reference LAD.

Route	Reference LAD MSDV $\left(m/s^{1.5}\right)$	Variable LAD MSDV $\left(m/s^{1.5}\right)$
Route 1	33.27	20.97
Route 2	15.87	11.09
Route 3	11.12	7.47

**Table 2:** Specifications of the simulation vehicle.

Autonomous Shuttle (KAMO_U)	
Driveline	Front Drive
Motor Power	91 kW
Max. Torque	310 Nm
Unload Weight	2500 kg
Length	5300 mm
Height	2500 mm
Wheelbase	3700 mm
Width	1735 mm
Rear overhang	800 mm
Tire	235/65 R17

**Table 3:** Simulation results of comparative control and motion sickness reduction control on the K-city evaluation route.

	Comparative Control	Motion Sickness Reduction Control
MSDV ($m/s^{2}$)	16.3	10.46
Travel Time (s)	300.2	295
Max Cross-Track Error (m)	0.96	0.776

## Data Availability

The data presented in this study are available upon request from the corresponding author.
